# Factors associated with poor outcome in right heart endocarditis

**DOI:** 10.1186/1471-2334-13-S1-P108

**Published:** 2013-12-16

**Authors:** Mihaela Lavinia Zamfira, Șerban Benea, Cozmina Andrei, Georgeta Ducu, Daniela Camburu, Mihaela Ionică, Alina Cozma, Roxana Dumitriu, Otilia Elisabeta Benea

**Affiliations:** 1 National Institute for Infectious Diseases “Prof. Dr. Matei Balş”, Bucharest, Romania; 2 Carol Davila University of Medicine and Pharmacy, Bucharest, Romania

## Backgroud

We studied factors associated with poor outcome in right heart endocarditis.

## Methods

In the period January 2011 – December 2012 at the National Institute for Infectious Diseases “Prof. Dr. Matei Balş” were admitted 53 patients with right endocarditis, with 72 episodes.

## Results

Males predominated (64.2%), patients in the age group 20-39 years (83%), those from urban areas (81%) and unemployed persons (69.8%). The infection was localized to the tricuspid valves – 42 cases, tricuspid valves plus left heart – 6 cases, right atrial wall – 3 cases, pulmonary valve – 1 case, right atrial device – 1 case. The main risk factor for right endocarditis was as IV drug use (86.8%). 87% of patients had HCV infection and 52.2% were HIV infected. Blood cultures were positive in 73.6% of cases. *Staphylococcus aureus* was the most frequently isolated (73.7%), type MSSA in 51.3% of cases.

Under treatment with antibiotics, anticoagulants, diuretics evolution was towards improvement in 52.8% of cases and 18.9% for death.

Factors associated with risk of poor outcome were: the presence of tricuspid murmurs (from 8/10 deaths vs. 13/43 survivors, p=0.003; OR=9.231, 95%CI: 1.719-49.55), the occurrence of embolic complications (4/10 deaths vs. 5/43 survivors, p=0.03; OR=5.067, 95%CI: 1.052-24.39), the presence of multiple pulmonary microabscesses (8/10 patients vs. 14/43, p=0.004; OR=8.286, 95%CI: 1.551-44.26), tricuspid vegetations larger than 10 mm (7/10 deaths vs. 5/43 survivors, p=0.0002; OR=17.73, 95%CI: 3.431-91.66), the association of HIV infection with elevated HIV-RNA and severe immune deficiency with CD4 below 200 cells/cmm.

